# Augmenting the logrank test in the design of clinical trials in which non-proportional hazards of the treatment effect may be anticipated

**DOI:** 10.1186/s12874-016-0110-x

**Published:** 2016-02-11

**Authors:** Patrick Royston, Mahesh K.B. Parmar

**Affiliations:** MRC Clinical Trials Unit at UCL, Aviation House 125 Kingsway, London, WC2B 6NH UK

**Keywords:** Time-to-event data, Randomized controlled trials, Hazard ratio, Restricted mean survival time, Non-proportional hazards, Logrank test, Permutation test, Design, Simulation, Flexible parametric model

## Abstract

**Background:**

Most randomized controlled trials with a time-to-event outcome are designed assuming proportional hazards (PH) of the treatment effect. The sample size calculation is based on a logrank test. However, non-proportional hazards are increasingly common. At analysis, the estimated hazards ratio with a confidence interval is usually presented. The estimate is often obtained from a Cox PH model with treatment as a covariate. If non-proportional hazards are present, the logrank and equivalent Cox tests may lose power. To safeguard power, we previously suggested a ‘joint test’ combining the Cox test with a test of non-proportional hazards. Unfortunately, a larger sample size is needed to preserve power under PH. Here, we describe a novel test that unites the Cox test with a permutation test based on restricted mean survival time.

**Methods:**

We propose a combined hypothesis test based on a permutation test of the difference in restricted mean survival time across time. The test involves the minimum of the Cox and permutation test P-values. We approximate its null distribution and correct it for correlation between the two P-values. Using extensive simulations, we assess the type 1 error and power of the combined test under several scenarios and compare with other tests. We investigate powering a trial using the combined test.

**Results:**

The type 1 error of the combined test is close to nominal. Power under proportional hazards is slightly lower than for the Cox test. Enhanced power is available when the treatment difference shows an ‘early effect’, an initial separation of survival curves which diminishes over time. The power is reduced under a ‘late effect’, when little or no difference in survival curves is seen for an initial period and then a late separation occurs. We propose a method of powering a trial using the combined test. The ‘insurance premium’ offered by the combined test to safeguard power under non-PH represents about a single-digit percentage increase in sample size.

**Conclusions:**

The combined test increases trial power under an early treatment effect and protects power under other scenarios. Use of restricted mean survival time facilitates testing and displaying a generalized treatment effect.

## Background

As we recently discussed [1], the design of almost all randomized controlled trials (RCTs) with a time-to-event outcome depends on the proportional hazards assumption for the treatment effect. However, for various reasons non-proportional hazards (non-PH) are being detected more frequently nowadays. The reasons may include larger trials which increase power to detect non-PH, and the development of new therapies with different modes of action.

The sample size calculation for such a trial assumes a logrank test will be performed. Under PH, the logrank test is closely equivalent to a Cox test, that is, a test based on the difference in log partial likelihoods with the binary treatment variable as the only covariate in a Cox PH model. In what follows, statements referring to the Cox test are equally applicable to the logrank test when PH holds. This may not be the case under non-PH. At the analysis stage, an estimate of the hazard ratio (HR) with a confidence interval (CI) is often obtained from the aforementioned Cox model. If non-PH is present, the HR varies over follow-up time, and the overall estimated HR that is obtained is a type of average over the event times [2].

To accommodate possible non-PH, we suggested [1] combining the Cox test with the Grambsch-Therneau test of non-PH [3] for a possible time-dependent treatment effect. We called it a ‘joint test’. We showed that the joint test has more power than the Cox test when certain patterns of non-PH are present. However, to obtain the same power as the Cox and logrank tests when PH holds, a substantial increase in the sample size is needed. For example, for a certain configuration of patient accrual and follow-up, to achieve power 90 *%* at a 5 *%* significance level in a two-arm trial with equal allocation, the Cox and logrank tests require 763 patients (509 events) whereas the joint test needs 919 patients (613 events), an increase of more than 20 *%*. A 20 *%* larger trial may be a heavy ‘insurance premium’ to guard against loss of power with some types of non-PH.

A second disadvantage of the joint test concerns the estimand. The Cox and logrank tests are most powerful (indeed, optimal) with a time-fixed hazard ratio, whereas the joint test responds to some fairly general patterns of non-PH. No estimand, readily interpretable in terms of a treatment effect, is available with the joint test. (In fact, the test statistic of the Grambsch-Therneau test is derived from a correlation between the scaled Schoenfeld residuals and the failure times. Under PH the population correlation is zero).

Recently, we have argued [4] for the restricted mean survival time (RMST) as a helpful and interpretable general measure of the treatment effect on the scale of time rather than survival probability or hazard. RMST may be used irrespective of whether PH holds or not. The RMST may be succinctly described as the mean survival time from randomization to a clinically relevant time horizon, *t*^∗^. The treatment effect is the change (usually, gain) in RMST at *t*^∗^ for the research treatment compared with control. The choice of a representative *t*^∗^ is context-dependent; typically it will be towards the end of the follow-up period when the trial data are most mature.

Mathematically, the RMST at *t*^∗^ equals the integrated survival function on (0,*t*^∗^) [5]. A convenient ‘non-parametric’ estimator, based on the Kaplan-Meier survival curve, involves jackknife quantities [6]. The estimator has been implemented in software for Stata [7, 8] and R [9].

An ‘obvious’ test statistic for the treatment effect based on RMST is the square of the ratio of the RMST difference to its standard error. Under the global null hypothesis of identical survival curves, the test statistic has a chisquare distribution on 1 d.f. However, such an approach has a serious challenge. The value of *t*^∗^ would need to be prespecified in the trial protocol, but in the ensuing data, the selected *t*^∗^ may be suboptimal, leading to a loss of power. A single *t*^∗^ is too fragile. A more natural test could be based on the maximal squared ratio over a suitable range of values of *t*^∗^. Such a test would be much less likely to ‘miss something important’—particularly under non-PH, when the largest cumulative difference between the survival curves may appear at essentially any *t*^∗^.

In the present paper, we develop an approach to design and testing based on the idea of paying a modest ‘insurance premium’, consisting of a slightly larger sample size, against the possibility of non-PH, as mentioned above for the joint test. To do so, we describe a test which combines the Cox test with a test of the RMST difference. The latter involves testing the RMST difference at various *t*^∗^ values, together with a suitable adjustment of the resulting minimal *P*-value to allow for multiple testing and correlation with the Cox test *P*-value.

The structure of the paper is as follows. In section ‘Methods’, we describe the trial datasets we use for illustration and as the basis of fairly extensive simulation studies. We describe RMST and discuss its dynamic role in understanding a generalized treatment effect. We define our new combined test, which is based on analysis of the time-dependent RMST curve and on the Cox test. We then describe the use of simulation to assess the power and type 1 error of the combined and other tests. Finally, we suggest a simple way of powering a trial using the combined test. Section ‘Results’ gives the results of the simulation studies and summarizes our findings. Section ‘Discussion’ rounds up some important additional points.

## Methods

### Datasets

As a source of illustrative data, chiefly for the purpose of simulation, we use data from three randomized trials: GOG111 in advanced ovarian cancer [10], PATCH1 in recurrent cellulitis of the leg [11], and ICON7, also in ovarian cancer [12]. The outcomes used in these trials are overall survival, time to first recurrence and progression-free survival, respectively. Table 1 gives statistics on the treatment effect in the trials.

**Table 1:** Statistics for three randomized trials used in examples

Trial	*n*	*e*	HR (SE)	*P*_*LR*_	*P*_*Cox*_	*P*_*GT*_	*P*_*joint*_	*P*_*perm*_	*P*_*comb*_
GOG111	386	343	0.73 (0.08)	0.0037	0.0038	0.0061	0.00035	0.00041	0.00062
PATCH1	274	129	0.71 (0.13)	0.052	0.052	0.051	0.023	0.015	0.023
ICON7	1528	759	0.81 (0.06)	0.0041	0.0042	9×10^−13^	1×10^−13^	4×10^−9^	6×10^−9^

*n* = number of patients, *e* = number of events For further details, see the text

*P*_*LR*_ denotes the *P*-value from the logrank test. *P*_*Cox*_ and *P*_*GT*_ represent the *P*-values from the Cox test and Grambsch-Therneau test of non-PH, respectively. *P*_*joint*_ is the *P*-value from the joint test [1]. *P*_*perm*_ and *P*_*comb*_ are described in section ‘Combined test of the treatment effect’. As anticipated, *P*_*LR*_ and *P*_*Cox*_ are very similar. We do not study *P*_*LR*_ any further.

**Fig. 1:**
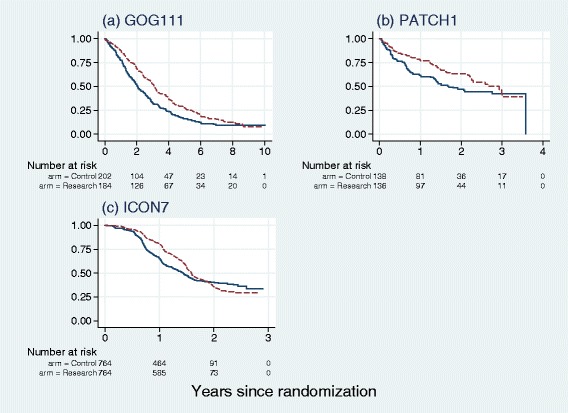
Kaplan-Meier plots for the three trials datasets. Solid line, control arm; dashed line, research arm

Figure 1 shows Kaplan-Meier plots of the datasets. All three trials exhibit a treatment effect (HR <1) and varying degrees of non-PH, although for the smaller PATCH1 trial, the *P*-values for the Cox and Grambsch-Therneau tests are just above the 5 *%* level (see Table 1). In ICON7 the non-PH is most marked and the Kaplan-Meier survival curves actually cross.

**Fig. 2:**
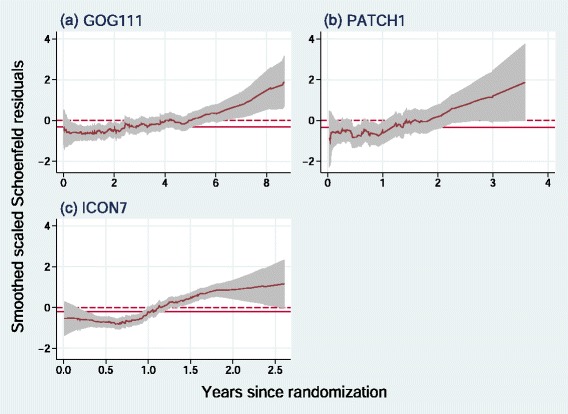
Smoothed scatter plots of scaled Schoenfeld residuals for the treatment variable in the three trials denoted by (**a**), (**b**) and (**c**). Grey shaded areas show pointwise 95 % confidence intervals. Solid horizontal lines show *y*= ln HR, the (constant) log hazard ratio estimated by a Cox model. Dashed horizontal lines show *y*=0, corresponding to ln(HR) =0, null treatment effect

Further information on the nature of possible non-PH in the three trials is provided by Fig. 2.

Scaled Schoenfeld residuals are the basis of the Grambsch-Therneau test; non-random trends with time (*t*) may indicate non-PH. Scatter plot smoothing is essential since the raw residuals invariably have considerable ‘noise’. Smoothed scaled Schoenfeld residuals are not necessarily an unbiased estimate of a time-dependent log HR [13], so the plots should be interpreted conservatively. In each trial there seems to be a positive treatment effect (ln HR <0) during the first part of follow up, diminishing over time and possibly switching to a negative treatment effect (ln HR >0) at longer follow up times. We describe this important phenomenon as an ‘early effect’ of treatment.

### Restricted mean survival time (RMST)

The RMST *μ* of a survival-time random variable *T*>0 is defined as the mean of min(*T,t*^∗^), where *T* is truncated at some horizon *t*^∗^>0. When *T* is years to death, we may describe *μ* as the ‘ *t*^∗^-year life expectancy’. RMST has been used to summarize survival outcomes when non-PH has been observed [14].

It can be shown that *μ* equals the area under the survival curve *S*(*t*) from 0 to *t*^∗^ [5, 15], that is

(1) $$ \mu=E\left[\min\left(T,t^{\ast}\right)\right] =\int_{0}^{t^{\ast} }S\left(t\right) dt   $$

In a two-arm clinical trial with survival functions *S*_0_(*t*) in the control arm and *S*_1_(*t*) in the research arm, the restricted gain in life expectancy, that is the difference in RMST between arms, is given by $\int _{0}^{t^{\ast }}S_{1}\left (t\right) dt-\int _{0}^{t^{\ast }}S_{0}\left (t\right) dt=\int _{0}^{t^{\ast }}\left [S_{1}\left (t\right) -S_{0}\left (t\right)\right ] dt$. Thus the gain is the (signed) area between the survival curves. Note that if the survival curves cross at some *t*<*t*^∗^ the RMST difference could change sign.

### RMST as a function of time

**Fig. 3:**
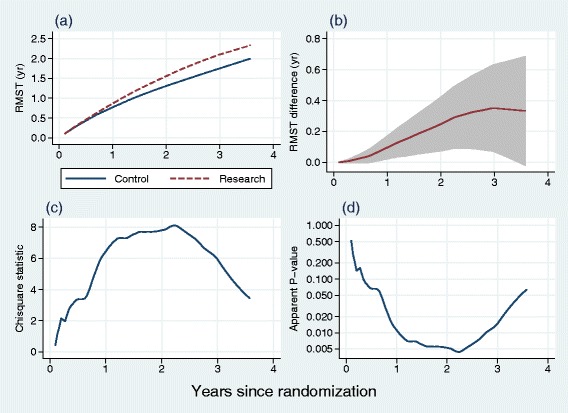
RMST analysis of the PATCH1 trial. All statistics are displayed as functions of time in years since randomization. **a** RMST in each treatment group; **b** RMST difference between treatment groups with 95 % pointwise confidence interval; **c** Chisquare statistics corresponding to (**b**); **d** *P*-value derived from values in (**c**)

Just like the survival function *S*(*t*), rather than focusing on a single *t*^∗^, RMST may be considered to be a function of time. As an example, we use the PATCH1 trial. The Kaplan-Meier curves by treatment arm are shown in Fig. 1 (b). Other functions related to RMST are shown in Fig. 3. As noted in Table 1, using conventional levels of statistical significance there is a borderline advantage of the research treatment (*P*=0.052, Cox test). Also, borderline significant non-PH is present (*P*=0.051, Grambsch-Therneau test). Further investigation shows that the log HR gets nearer to 0 over time, and may even exceed 0 at longer follow-up times (see Fig. 2 (b)).

Figure 3 (a) shows the RMST as a function of time at several time points in each arm, and (b) the difference in RMST with a 95 % pointwise confidence interval, calculated up to the largest event time (3.57 years). The RMST difference increases over time up to 3 years. The maximal chisquare statistic is 8.11 and occurs at *t*=2.22 years. As shown in Fig. 3 (b), the standard error of the RMST difference increases over time; this is because the number of patients still at risk diminishes, mainly due to administrative censoring. The apparent *P*-value for the RMST difference (see Fig. 3 (d)) is 0.0044, seemingly highly significant and much smaller than the Cox test *P*-value. However, the apparent *P*-value is invalid as it does not allow for multiple testing. Nevertheless, the RMST analysis suggests an important difference in time to recurrence may be present in the data, peaking at around 3 years (see Fig. 3 (b)). The example motivates further investigation of the maximal chisquare statistic, as follows.

### A permutation test for the maximal chisquare statistic

Theoretically, no test of two survival curves can have higher power than the logrank and Cox tests when PH holds. However, if the data exhibit evidence of non-PH, the power of the tests may be reduced and a test based on the maximal RMST chisquare statistic might do better.

If there is an important difference between the survival curves for the treatments over some region of the time axis, the RMST difference should reflect it. To locate such a region, we consider searching over a suitable grid of times to find the *t*^∗^ value that maximizes the chisquare statistic, *X*^2^, for testing the RMST difference. Call the maximal chisquare value *C*_max_. When there is a real treatment difference, *C*_max_ should be ‘large’ and ‘significant’.

Because the search for *C*_max_ by definition involves multiple testing of the RMST difference, the *P*-value for *C*_max_ derived from the chisquare distribution on 1 d.f. is too small and gives an inflated type 1 error in the global null case of identical population survival curves. To overcome the difficulty, we adopt a permutation test approach to obtain a corrected *P*-value for *C*_max_. The aim is to determine how extreme is *C*_max_ within the null distribution of the maximal chisquare statistic. For example, if *C*_max_ lay at the 95th centile of the null distribution, the corrected *P*-value would be 0.05.

In practice, such a permutation test requires *a priori* choices of a suitable interval over which to vary *t*^∗^ and of the number (*n*_*t*_) of *t*^∗^ values at which to determine *C*_max_. In many trials, it is reasonable to argue that a proportion of the frailest patients will succumb to an event fairly early on, irrespective of the treatment they receive. Furthermore, we are unlikely to obtain a reliable, representative and clinically meaningful estimate of RMST difference early in follow-up. A sensible choice for the lower bound of the interval should therefore not be too small. We chose the 30th centile of the event times as our preferred lower bound.

Under PH, the RMST difference continues to increase with *t*^∗^. A logical choice for the upper bound is therefore the largest (uncensored) event time. Note that when using the jackknife method, RMST cannot be estimated beyond the largest event time anyway.

The choice of *n*_*t*_, the number of time points at which RMST and *X*^2^ are evaluated, is somewhat arbitrary. In 20 non-randomly chosen trials datasets in our keeping, mostly in cancer, we compared the *P*-values for the permutation test resulting from *n*_*t*_=5, 10, 15 and 20 and found that 5 tended to miss the optimal *t*^∗^ too often. There was little to choose between 10, 15 and 20. For economy of computation we chose *n*_*t*_=10.

To operationalize the test, we randomly permute the treatment covariate a large number *M* of times. This removes any systematic association between the outcome and the treatment assignment while preserving the remaining structure of the survival data. In each permuted dataset we determine the maximal chisquare statistic over the *n*_*t*_=10 selected equally spaced times, giving a sample *C*_1_,…,*C*_*M*_ drawn from the null distribution of *C*_max_. Let $N=\sum _{i=1}^{M}I\left (C_{i}>C_{\max }\right)$ be the number of permutation samples in which *C*_*i*_ exceeds *C*_max_, where *I*(.) is the indicator function. For example, if *C*_max_ was larger than all the *C*_*i*_ then *N*=0. *N* has a binomial distribution with denominator *M*. The *P*-value for the permutation test is determined as *P*_*perm*_=(*N*+0.5)/(*M*+1), where 0.5 is a continuity correction. The resolution of the test, that is the ‘most significant’ *P*-value available with a given *M*, is 0.5/(*M*+1).

Let *P*=*N*/*M*. By simple algebra, the variance of *P*_*perm*_ equals [*M*/(*M*+1)]^2^*var*(*P*)=[*M*/(*M*+1)]^2^[*P*(1−*P*)/*M*]. We are free to choose *M*. How large should *M* be? For performing simulation studies, which are compute-intensive for large *M*, we suggest using *M*=999, giving a resolution of 0.0005. For definitive data analysis, a much larger value of *M* may be required.

### Approximating the permutation test

As defined above, the permutation test is plausible but with three noteworthy drawbacks: (i) *P*_*perm*_ is stochastic, meaning that it has a chance component and is not precisely reproducible (different analysts will get different answers for the same dataset); (ii) an appropriate choice of *M* is not ‘obvious’; and (iii) simulation studies of the test may consume considerable computer time. A simple approximation to the test would be valuable.

**Fig. 4:**
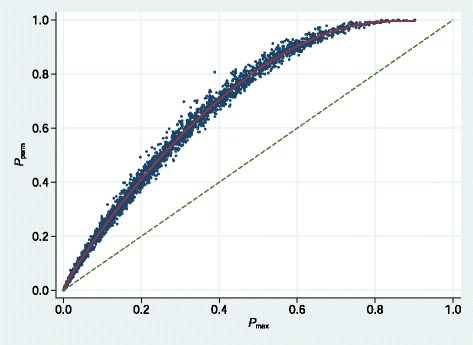
Scatter plot of *P*_*perm*_ against *P*_max_ in 3000 simulated samples based on 3 datasets. Solid curve shows an empirical fit from a Box-Tidwell model. Diagonal dashed line is the line of identity

We hypothesized that *P*_*perm*_ must bear a strong relationship with *P*_max_, the uncorrected *P*-value from *C*_max_. We simulated 1000 replicates of the global null case in each of the three example datasets and computed *P*_*perm*_ (with *M*=999) and *P*_max_ in each of the 3000 samples. A plot of *P*_*perm*_ against *P*_max_ is shown in Fig. 4.

The large degree of ‘optimism’ in *P*_max_ is clearly visible. After trial and error, we fitted a Box-Tidwell model of the form $E\left (y\right) =\beta _{1}x^{p_{1}}+\beta _{2}x^{p_{2}}$, with *p*_1_ and *p*_2_ real numbers estimated from the data, to obtain the following approximation to *P*_*perm*_:

(2) $$ E\left(P_{perm}\right) =1.762\left(P_{\max}\right)^{0.885}-0.802\left(P_{\max}\right)^{2.547}   $$

To avoid a tiny downturn in *E*(*P*_*perm*_) for *P*_max_>0.85, we truncated the fitted curve at (*P*_*perm*_,*P*_max_)=(0.9963,0.85). As can be seen in Fig. 4, the fit is excellent throughout the range of *P*_*perm*_. We used the approximation (2) in subsequent work, so that *P*_*perm*_ now denotes the approximate permutation test *P*-value calculated from *P*_max_.

In the Appendix, we study the accuracy and generalizability of the approximations (2) and (3) through the type 1 errors of *E*(*P*_*perm*_) and *P*_*comb*_ through simulations based on the twenty trials mentioned in section ‘A permutation test for the maximal chisquare statistic’. We find a small amount of non-random variation in the empirical type 1 errors. Overall, however, the variations do not appear large enough to be of practical concern, and the mean empirical type 1 errors are close to their nominal values. Note that the original version of *P*_*perm*_ can be calculated as a check on the approximation in critical cases, e.g. when the approximated *P*-value is near an important cutoff such as 0.05.

### Combined test of the treatment effect

Next, we consider a new test which combines the Cox test with the permutation test. The aim is to assess the strengths of each test across a range of alternatives. We calculate *P*_min_, the smaller of the two *P*-values,

$$P_{\min}=\min\left(P_{Cox},P_{perm}\right) $$

where *P*_*perm*_ is shorthand for *E*(*P*_*perm*_) in (2). Although under the null hypothesis *P*_*Cox*_ and *P*_*perm*_ are each uniformly distributed, they are positively correlated, since each responds to departures from the global null. As a result, *P*_min_ will be ‘significant’ too often. We adjust *P*_min_ to allow for the correlation to obtain a test with the correct type 1 error probability.

We addressed adjustment using simulation followed by approximation of the null distribution of *P*_min_. Based on the three trials datasets described above, we simulated realistic replicates of the datasets using the Stata program stsurvsim [16], as outlined in section ‘Approach to simulation’. In each replicate of each dataset, the treatment covariate was randomly assigned to 0 or 1 with 1:1 allocation. We obtained null-distribution values of *P*_*Cox*_ and *P*_*perm*_ and hence *P*_min_ in each of 10,000 replicates per dataset.

We approximated the null distribution of *P*_min_ empirically. Because its support is confined to the interval (0,1) (appropriate for a *P*-value) and it is fairly flexible, we modelled *P*_min_ using a two-parameter beta distribution. Its cumulative distribution function is the incomplete beta function

$$I\left(P_{\min};a,b\right) =\frac{\Gamma\left(a+b\right)}{\Gamma\left(a\right) \Gamma b}\int_{0}^{P_{\min}}x^{a-1}\left(1-x\right) ^{b-1}\text{d}x $$

We estimated the parameters *a* and *b* by maximum likelihood. The estimate of *a* was close to 1 in each dataset. For simplicity, we constrained *a*=1. The estimates of *b* in the three datasets were very similar. We therefore pooled the data and estimated a single value, $\widehat {b}=1.51$ (95 % CI =1.49 to 1.53). We used the rounded value *b*=1.5 in subsequent work. Thus, to calculate *P*_*comb*_ from a given *P*_min_ we apply the formula

(3) $$ P_{comb}=I\left(P_{\min};1,1.5\right)   $$

Note that as *P*_min_→0 the limiting value of *I*(*P*_min_;1,*b*)/*P*_min_ equals *b*. In effect, for small *P*_min_ the adjusted *P*-value is *P*_min_/*b*. If *P*_*Cox*_ and *P*_*perm*_ were independent, the Bonferroni correction would apply and a similar analysis would give *b*=2. With *b*=1.5, we are taking advantage of the correlation and improving on the conservative Bonferroni correction.

The value of *P*_min_ corresponding to a given value of *P*_*comb*_ is given by inverting (3) via the inverse incomplete beta function,

$$P_{\min}=I^{-1}\left(P_{comb};1,1.5\right) $$

For example, the 0.05 significance level for *P*_*comb*_ is equivalent to the 0.0336 significance level for *P*_min_.

### Approach to simulation

We aimed to make the simulations of the type 1 error and power of the various tests as realistic as possible. To this end, we chose the three randomized trials described in section ‘Datasets’ as prototypes, and mimicked sampling from the distribution of time to event in each dataset under four different scenarios (see section ‘Power of the combined test’ for details of the scenarios). In general terms, we first fitted a flexible parametric model, without covariates, to each dataset separately and estimated the parameters of a restricted cubic spline function with 3 degrees of freedom (d.f.) used to approximate the log cumulative hazard function. We then reversed the event indicator and estimated the distribution of the time to censoring by the same approach. Using the Stata package stsurvsim [16], we simulated times to event according to the survival distributions represented by the estimated log cumulative hazard functions. We censored the times to event as necesary by also simulating times to censoring, using the second set of spline parameter estimates. In this way, we were able to produce realistic replicates of the trial datasets with an appropriate amount of stochastic variation induced.

We approached simulation of particular treatment effects as follows. Under the global null (scenario A, see section ‘Power of the combined test’) we simulated a complete dataset of the required sample size (*n*) and divided it at random into two equal subsamples, thus defining a treatment variable with no effect, coded 0= control arm, 1= research arm. Under PH (scenario B) we simulated the control arm as for scenario A. For the research arm, we modified the spline function representing the log cumulative hazard function from the overall data by adding ln(0.75), meaning that the underlying hazard function in the research arm was to be 0.75 times that in the control arm. We simulated data from the modified research arm spline function and concatenated the two subsamples to create a single simulation replicate with a PH treatment effect.

In scenario C (putative early effect) we ‘let the data speak’. We simulated from the control and research arms in the original data from the three trials independently of one another according to separate flexible parametric models. This enabled us to produce simulation replicates realistically similar to the original trial datasets, all of which exhibited an early effect (see Fig. 2).

**Fig. 5:**
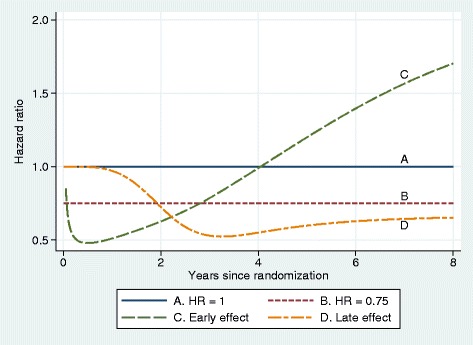
Hazard ratio functions used in scenarios A, B, C and D

In scenario D we induced the late effect artificially, in principle as done in scenario B. Because of its relatively long follow up time, this was done only for the GOG111 dataset. We simulated the control arm as in scenario B and modified the log cumulative hazard function in such a way as to produce an HR function that was 1 up to one year (no treatment effect) and <1 at later times (beneficial late effect of the research treatment). A between-arm difference in the log cumulative hazard functions was induced by applying a decreasing logistic function of log time to the control arm function. The resulting hazard ratio function was calculated mathematically. See Fig. 5 for an illustration of the four hazard ratio functions that underlie datasets simulated from the GOG111 trial data.

### Power of the combined test

As mentioned above, we studied four scenarios (A–D) for assessing type 1 error and power: A. global null with HR = 1; B. proportional hazards with HR =0.75; C. non-PH with an early treatment effect dwindling over time (as seen in all the example datasets); and D. late treatment effect beginning after 1 year. Simulation scenarios A–C were based on all three datasets, whereas scenario D was applied to the GOG111 dataset only.

**Fig. 6:**
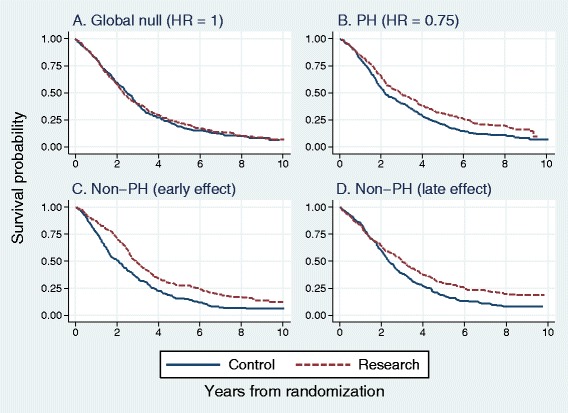
Kaplan-Meier survival curves illustrating the treatment effect in one arbitrarily chosen simulation replicate of scenarios A–D, each with sample size 500. Survival pattern in the control arm and censoring pattern are derived from the GOG111 trial dataset

We illustrate the type of data that arises in the simulation. Figure 6 shows Kaplan-Meier curves of the treatment effect in one arbitrarily chosen replicate in each scenario based on the GOG111 dataset.

The nature of departures from PH can be quite subtle and not easy to recognize from Kaplan-Meier plots, which are the most frequent way of displaying survival data. Further insight is provided by Fig. 5, which shows how different are the hazard ratio functions for scenarios A–D.

Note how difficult it is to distinguish ‘by eye’ between the survival patterns in scenarios B and C. Yet, as we shall see, the logrank and Cox tests perform differently between these situations. Other tests may be more powerful than Cox/logrank tests in scenario C, which is not the case in scenario B.

In each of scenarios B, C and D, 5,000 independent replicated datasets were generated, whereas in scenario A, 10,000 replicates were created as part of the type 1 error investigation already discussed. In scenarios B, C and D, sample sizes (*n*) were chosen by trial and error to provide power of about 90 *%* for the combined test, which thereby served as a benchmark for assessing the other tests. The sample size used for scenario A was essentially arbitrary; we took *n*=1000.

### Applying the combined test to trial design

We intend the combined test to offer an ‘insurance policy’ against possible loss of power under non-PH, particularly in the case of an early effect—a treatment effect whose HR favours the research arm (i.e. HR <1) early in the trial and approaches or even exceeds 1 over time. When PH holds, the ‘insurance premium’ requires a small increase in sample size, as described in the following example.

We consider an example of a design scenario, based this time on advanced bladder cancer, in which the survival function in the control arm over 1,2,…,12 years is assumed to be 0.767, 0.628, 0.529, 0.453, 0.392, 0.343, 0.302, 0.268, 0.238, 0.213, 0.191, 0.172. We suppose patient recruitment at a uniform rate over 8 years, with follow-up for a further 4 years. According to ART trial design methodology [17, 18], powering the logrank/Cox test under PH at 90 *%* for significance level *α*=0.05 requires 763 patients with 509 events. For the *P*_min_ cutoff of *α*=0.0336 (giving cutoff 0.05 for *P*_*comb*_), *n* increases by 10.6 *%* to 843 patients (562 events). Simulation (detail not reported) suggests that the power of the combined test, designed as though it was a logrank/Cox test with *α*=0.0336, is about 91 *%* (1 *%* above nominal). This shows that the increase in sample size from 763 to 843 somewhat overshoots what is needed to achieve power 90 *%*. A simple correction is to power the combined test at 89 *%* with *α*=0.0336 rather than 90 *%*. This requires 816 patients (544 events), a small but potentially worthwhile reduction in trial resources.

We do not advocate attempting to power a trial according to the logrank/Cox test under PH alone or under a particular prespecified pattern of non-PH. The assumed functional form for the time-dependent HR may be seriously in error, with unforeseeable consequences for power. As will be apparent in the results of the simulation studies described below, the Cox test continues to provide reasonable power in some non-PH settings. However, its power can be severely reduced in the case of an early effect. As in our earlier paper [1], we propose to power a trial under PH, accommodating an ‘insurance premium’ by taking the significance level for the Cox test to be the *P*_min_ that achieves *P*_*comb*_=0.05. As already stated, to implement a design with *α*=0.05 and power approximately 90 *%* for the combined test, the significance level for the logrank and Cox tests needs to be *α*=0.0336 and the power 89 *%*.

### Display of data, estimation and testing

We recommend plots resembling Figs. 1, 2 and 3 to display the data, estimation of RMST and RMST difference, possible pattern of non-PH and preliminary testing for RMST difference. The RMST difference at any given *t*^∗^ can be regarded in Fig. 3 (b) as an instantaneous value of the continuous function and can be presented separately with its 95 % CI. Corrected significance testing of maximal standardized RMST difference requires calculation of *P*_*perm*_, the approximate permutation test. Testing the treatment effect according to the methods proposed in this paper also requires the Cox test and the resulting values of *P*_min_ and *P*_*comb*_.

## Results

### Simulations

In Table 2, we report the results of the simulation studies based on data from the three randomized trials (GOG111, PATCH1, ICON7) in the four scenarios studied (see Fig. 6).

**Table 2:** Results of simulation studies

Scenario	Dataset	*n*	Test			
			Cox	Joint	Perm.	Comb.
A (null)	GOG111	1000	5.2	5.3	4.9	5.3
	PATCH1	1000	5.0	4.9	5.2	4.8
	ICON7	1000	4.8	4.9	5.2	4.9
B (PH)	GOG111	652	**9** **2** **.** **9**	87.2	*8* *6*.*7*	91.0
	PATCH1	1280	**9** **2** **.** **6**	*8* *6*.*9*	87.7	90.2
	ICON7	1240	**9** **1** **.** **9**	*8* *6*.*6*	88.3	89.8
C (early)	GOG111	310	*7* *2*.*5*	91.5	**9** **2** **.** **1**	90.0
	PATCH1	450	*7* *4*.*4*	88.8	**9** **1** **.** **9**	89.2
	ICON7	522	*3* *6*.*9*	**9** **8** **.** **7**	92.4	89.5
D (late)	GOG111	560	92.7	**9** **6** **.** **1**	*8* *0*.*5*	90.3

Values in table are percentages of 10,000 (scenario A) or 5,000 (scenarios B–D) simulated datasets in which each of four tests was significant at the 5 percent level. The datasets were simulated to mimic data from three randomized controlled trials with varying sample size (*n*). Values in bold (or italic) type indicate the most (or least) powerful among the four tests for the given scenario and dataset

Abbreviations: *Joint* joint test [1], *Perm*. permutation test, *Comb*. combined test

The first three rows report the estimated type 1 error at a nominal significance level of 5 *%* for each test. The three rows labelled B (PH) give the estimated power under PH (scenario B in Fig. 6), with target HR =0.75. The remaining four rows show the estimated power under the C (early) and D (late) effect scenarios.

### Summary of findings

1. Type 1 error: consistent with the nominal 5 *%* value for all tests and datasets.
2. Power under proportional hazards: as expected, the Cox test has slightly more power (about +2 *%*) than the combined test. The joint test is weaker than both the combined test (power about −3.5 *%*) and the Cox test (−5.5 *%*).
3. Power under early effect: the Cox test can suffer a dramatic lack of power in this type of scenario (note particularly ICON7). The joint test can perform very well when marked non-PH is present, as in ICON7. The combined test also performs well in the early effect scenarios.
4. Power under late effect: the joint test is the most powerful in this example. The combined test performs slightly worse than the Cox test (−2.4 *%*).
5. Overall, although sometimes performing well, the permutation test is not powerful enough to be recommended as the sole test.

In summary, the combined test performs well in all scenarios and is only a little (not critically) weak under PH; power is about 1.9 *%* to 2.4 *%* lower than the Cox test. With an early treatment effect, the combined test can be dramatically more powerful than the Cox test. Although never the most powerful among the four tests considered in the case studies, the combined test is nevertheless recommendable as an ‘omnibus test’ of a generalized treatment effect. The joint test generally performs well in the non-PH scenarios but, critically, it is rather weak under PH. This is because the non-PH component of the joint test is essentially ‘wasted’ under PH.

## Discussion

Many tests comparing two survival distributions have been proposed and studied. For example, Li et al. [19] recently compared the power of 21 such tests in relatively small samples in which the survival curves cross. With the power of the logrank test as reference, all of the tests considered by Li et al. [19] exhibited mild to severe loss of power under PH. Working in the context of randomized controlled trials with a time to event outcome, we have developed what we believe to be an acceptably ‘omnibus’ combined test of a generalized treatment effect based on the Cox/logrank test and the maximal squared standardized difference in RMST. Earlier, we proposed [1] a similarly motivated joint test comprising the sum of the Cox partial likelihood ratio statistic and the chisquare for the Grambsch-Therneau test of non-PH. The present combined test is somewhat more complex to compute than the joint test, and does not outperform the joint test in some of the simulation scenarios we considered. If we accept augmentation of the Cox/logrank test as a reasonable strategy to protect power under non-PH and support sample size and power calculations for the design of trials under PH, why should we prefer the combined test to the joint test?

There are two main reasons. First, past experience suggests that in a proportion of trials the PH assumption holds, at least approximately. In such cases, the Grambsch-Therneau test of non-PH will have very low power and correspondingly the power of the joint test will be reduced. The expectation is confirmed by our simulation studies (see Table 2, scenario B). Second, the joint test is not associated with any identifiable estimate of the treatment effect or its behaviour over time. The permutation test and hence the combined test reflect the behaviour of the standardized difference in RMST over time (see Fig. 3).

Inspection of the two components (*P*_*perm*_ and *P*_*Cox*_) of the combined test may indicate which is the dominant feature. If *P*_*Cox*_<*P*_*perm*_ then the treatment effect is more likely to be approximately PH. If *P*_*perm*_<*P*_*Cox*_ then non-PH is more likely to dominate. In any case, further information may be obtained from the Grambsch-Therneau test and from smoothed scatter plots of scaled Schoenfeld residuals, such as Figs. 1 and 3. In all the example trials (see Table 1) we observe *P*_*perm*_<*P*_*Cox*_, suggesting that the non-PH elements are important here. Note that for PATCH1 the combined test is significant at the 5 *%* level (*P*_*comb*_=0.023) whereas the Cox test is not (*P*_*Cox*_=0.052). The two tests might therefore lead an analyst using conventional levels of statistical significance to draw different conclusions.

Our proposed strategy for trial design based on the combined test, to provide power 90 *%* at a two-sided significance level of 5 *%* under PH, is to power the logrank or Cox tests for 89 *%* power at significance level 0.0336. An advantage of this approach is that the various tools that are available for refining the design to reflect accrual rate and pattern, loss to follow up, time to recruit and time for follow up can all be used essentially without modification. In the example based on GOG111, such a design will require an ‘insurance premium’ of only about 7 *%* more patients and events than one based on a logrank or Cox test with power 90 *%* at *α*=0.05. The main gain is a considerable improvement in power if an early effect of treatment occurs which then dwindles over time. Such an enhancement is potentially valuable when the overall HR lies between the target value and 1 and the power of the logrank and Cox tests may be correspondingly low.

An alternative strategy that might appeal to some is to design the trial according to one of the many variants of weighted logrank tests that are available [20, 21]. For example, if an early effect is anticipated, power might be increased by assigning higher weights to early events and lower weights to later events, and *vice versa* for a late effect. Comparison of the power of weighted logrank tests with that of the several tests exemplified here, further research beyond the present scope, may be of interest.

**Fig. 7:**
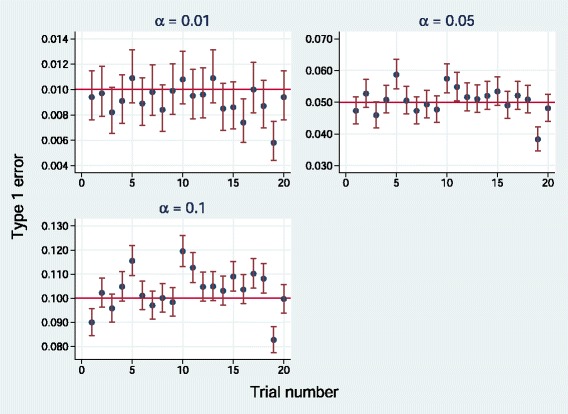
Empirical type 1 error of *E*(*P*_*perm*_) for simulations of 20 randomized trials datasets at three nominal significance levels. Error bars show 95 % pointwise confidence intervals

**Fig. 8:**
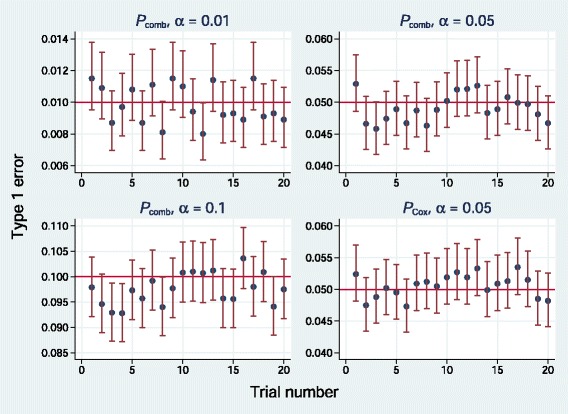
Empirical type 1 error of *P*_*comb*_ and *P*_*Cox*_ (*α*=0.05 only) for simulations of 20 randomized trials datasets at various nominal significance levels. Error bars show 95 % pointwise confidence intervals

In any case, three salient criticisms of a weighted logrank test approach may be made. First, crucially, as trial designers we have to prespecify the test before seeing the data. If we get the assumption of the pattern of non-PH wrong, for example assigning weights assuming a late effect when the ‘truth’ is an early effect, the resulting power could be disastrously low. The putative ‘insurance’ offered by our combined test, to try to guard against such mistakes, is important here. Second, in choosing the summary statistic, is a weighted hazard ratio or a standard hazard ratio to be used, and in any case how may either be interpreted in the light of non-PH? Third, the use of weights for event times in such a logrank test suggests that some events are ‘more important’ than others when calculating the result of a trial. Would such an emphasis be acceptable to consumers and investigators? We suspect not.

A reviewer pointed out that a permutation test may perform poorly in presence of confounders. Three comments may be made. First, by design of randomized controlled trials, the treatment effect is orthogonal to covariates, therefore confounding is not an important issue. Achieving orthogonality is indeed a key motivation for randomization. (Confounding could arise in trials that are too small and/or in which randomization has been botched.) Second, to our knowledge trials are not normally designed allowing adjustment for confounders. Thus if at all, confounding is an issue for analysis, not design. Although we are not convinced that adjustment is necessary in time-to-event trials, we are aware that some researchers do routinely adjust for stratification factors and sometimes for prognostically important covariates in the definitive analysis of their trial data. Third, note that in our approach the permutation test is only the starting point for the proposed combined test. Its main role is to establish the null distribution of the RMST-based test statistic, *C*_max_. We approximate the permutation test *P*-value through transformation (2) of *P*_max_. The permutation test result is not directly used in determining *P*_*comb*_.

In a recent report, Uno et al. [22] described an approach to hypothesis testing of the difference between two Kaplan-Meier survival curves. The concept is somewhat similar in spirit to our own proposal,though the details are quite dissimilar. Their two tests, *V*_1_ and *V*_2_, as described are one-sided. They are based on the integral over time of weighted, standardized differences between the survival functions. The power of the tests is studied by simulation under PH and under several different non-PH scenarios, akin to our own assessment. The power of the tests in the non-PH scenarios exemplified seems impressive, dominating that of other tests considered, including several flavours of the logrank. However, their performance under PH is less satisfactory. The power difference of *V*_1_ and *V*_2_ compared with the logrank test seems to depend on the shape of the survival curve that is being simulated (see their Fig. 6). In the best case the power of the logrank and new tests is about the same, whereas in the worst case, there can be as much as a ten percentage point difference. Plausibly reliable performance under PH is one reason we prefer our new combined test to our older joint test. Another drawback of Uno and colleagues’ proposal [22] is that the authors do not consider how to power a trial with one of their tests as the final analysis.

Of course, the credibility and robustness of a trial design, based on the combined test as an insurance against non-PH, depends on whether the test remains acceptably powerful when exposed to other realistic patterns of PH and non-PH survival curves. Using simulations, we have tried to cover common, plausible situations that we have seen in real trials. However, we acknowledge that stochastic simulation can only ever provide snapshots of a wide and varied landscape with local features that may be very different from those we choose to mimic in simulation designs. Further investigation of the performance of the combined test and other tests in a wider range of trials and accompanying simulations is a challenging topic for additional research.

Software for Stata to implement the various estimates and tests described in this article is under development and will be reported in the Stata Journal in due course.

## Conclusions

The combined test we propose increases trial power when an early treatment effect is present and protects power under other patterns of treatment effect, including proportional hazards. We recommend analytical and graphical use of restricted mean survival time to facilitate testing and displaying a generalized treatment effect. With minor modifications, standard methodology for trial design based on the logrank test can still be used with the combined test.

## Appendix

To check the accuracy and generalizability of the approximations (2) for *E*(*P*_*perm*_) and (3) for *P*_*comb*_, we investigated empirical type 1 errors in 10,000 simulation replicates of each of the 20 trials datasets mentioned in section ‘A permutation test for the maximal chisquare statistic’. Figure 7 shows results for *E*(*P*_*perm*_) at nominal significance levels (*α*) of 0.01, 0.05 and 0.1. With some minor heterogeneity, values are generally close to nominal. Mean type 1 errors are 0.0092, 0.050 and 0.103.

Figure 8 shows analogous results for *P*_*comb*_. For comparison, we have included *α*=0.05 results for *P*_*Cox*_. Again, results are close to nominal, overall means for *P*_*comb*_ being 0.0098, 0.049 and 0.098.

We conclude that the approximations required to estimate *P*_*perm*_ and *P*_*comb*_ are adequate for practical application.

## Abbreviations

CI: Confidence interval
d.f.: Degrees of freedom
HR: Hazard ratio
MRC: Medical Research Council
Non-PH: Non-proportional hazards
PH: Proportional hazards
RCT: Randomized controlled trial
RMST: Restricted mean survival time
